# Real spinal cord injury without radiologic abnormality in pediatric patient with tight filum terminale following minor trauma: a case report

**DOI:** 10.1186/s12887-019-1894-8

**Published:** 2019-12-23

**Authors:** Qin Chuan Liang, Bo Yang, Yun Hai Song, Pin Pin Gao, Ze Yang Xia, Nan Bao

**Affiliations:** Department of Neurosurgery, Shanghai Children’s Medical Center Affiliated to Shanghai Jiaotong University School of Medicine, Dongfang Road 1678, Shanghai, 200127 China

**Keywords:** Pediatric, SCIWORA, Spinal cord injury, Tight filum terminale, Tethered cord syndrome

## Abstract

**Background:**

Spinal cord injury without radiographic abnormality (SCIWORA) is defined as having “clinical symptoms of traumatic myelopathy with no radiographic or computed tomographic features of spinal fracture or instability”. The mechanism of pediatric SCIWORA following minor trauma is still unclear. Tight filum terminale (TFT) has been studied in the literature, but the information regarding the predisposing factor for SCIWORA is still being defined.

**Case presentation:**

We report three cases of thoracic and lumber SCIWORA with TFT. The trauma was potentially mild in all cases but resulted in catastrophic damage of the cord. All patients had no signs or symptoms of tethered cord syndrome prior to the minor trauma. TFT was found during operation.

**Conclusions:**

We suggest that TFT might be a predisposing factor for SCIWORA and chronic spinal cord traction play an important role in the mechanism of pediatric thoracic and lumber SCIWORA following minor trauma. Patients who never undergo treatment for TFT likely have an elevated risk of developing SCIWORA following minor trauma.

## Background

Spinal cord injury without radiographic abnormality (SCIWORA) was defined as a myelopathy with no radiographic or computed tomographic features of spinal fracture or instability [1]. This disorder is more common in children, accounting for 6 to 19% of all pediatric spinal cord injuries [2]. SCIWORA is more often associated with high-energy injuries in younger children [3]. In older children, especially adolescents, SCIWORA is most commonly associated with athletic competition or automobile accidents. The mechanism of pediatric SCIWORA following minor trauma is still unclear. The trauma factors of pediatric SCIWORA include hyperextension, flexion, distraction, direct crush injury or combinations and ischemia spinal cord injuries due to the unique and inherent anatomic malleability of the pediatric spine [4].

Tethered cord syndrome (TCS) refers to a group of signs and symptoms of motor and sensory neuron dysfunction attributable to spinal cord traction [5]. TCS is a component of occult spinal dysraphism, such as tight filum terminale (TFT) [6]. TFT has been well studied in the literature, but the information regarding the predisposing factor for SCIWORA is still being defined. We report such 3 cases of thoracic and lumber SCIWORA with TFT. To the best of our knowledge, this is the first report of SCIWORA with TFT. TFT was found during operation. We suggest that TFT and chronic spinal cord traction play an important role in the mechanism of SCIWORA following minor trauma.

## Case presentation

### Case one

**Fig. 1:**
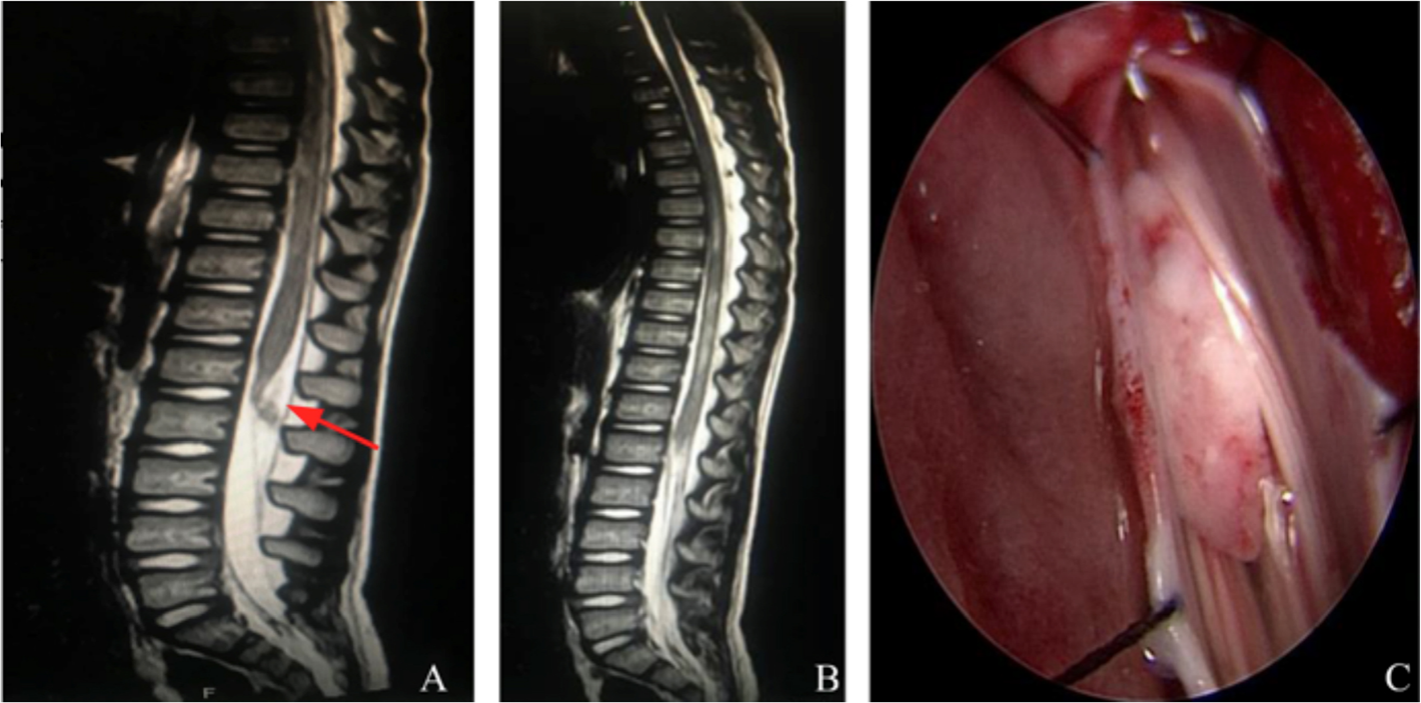
**a** A nodular T2 isointensity shadow was showed at the dorsal of the conus, which was located at L1 level with moderate swelling. **b** The sagittal MRI images showed longitudinally extended intramedullary patchy T2 hyperintensity at the T9-L1 level. **c** There were contusion and laceration in the cone and some contusion and laceration tissue outflew the soft spinal meninges

The patient was a 6-year-old girl who had no signs or symptoms of tethered cord syndrome prior to the minor trauma. Her father struck her hipshot with a painting pen and she performed evasive action with back bend movement. 30 min later, she could not get up again because of weakness in her legs. She complained of back pain and suffered from incontinence. On admission to the hospital 30 h after injury, she had paralysis of the lower extremities and dysfunction of two bowel movements. No signs of external wounds were found. She had no particular past history. A blood examination demonstrated no specific findings. Computed tomography (CT) of the spine showed spina bifida occulta. A spinal cord magnetic resonance imaging (MRI) performed 3 days following the injury. The sagittal MRI images showed longitudinally extended intramedullary patchy T1 hypointensity and T2 hyperintensity at the T9-L1 level. The conus was located at L1 level with moderate swelling. A nodular T1 isointensity and T2 isointensity shadow was showed at the dorsal of the cone. The next day, the patient underwent extended surgical exploration and lysis of the filum terminale. In the operation, we found 2 mm in diameter TFT with fibrous degeneration, which was confirmed by the histopathologic examination. There was contusion and laceration in the conus medullary. Some contusion and laceration tissue outflew the soft spinal meninges (Fig. 1). She remained in the hospital for 14 days. Upon discharge, the patient still complained of residual weakness and paresthesias in the lower extremities. By the 1-year follow-up, the patient still had paralysis of the lower extremities and dysfunction of two bowel movements.

### Case two

**Fig. 2:**
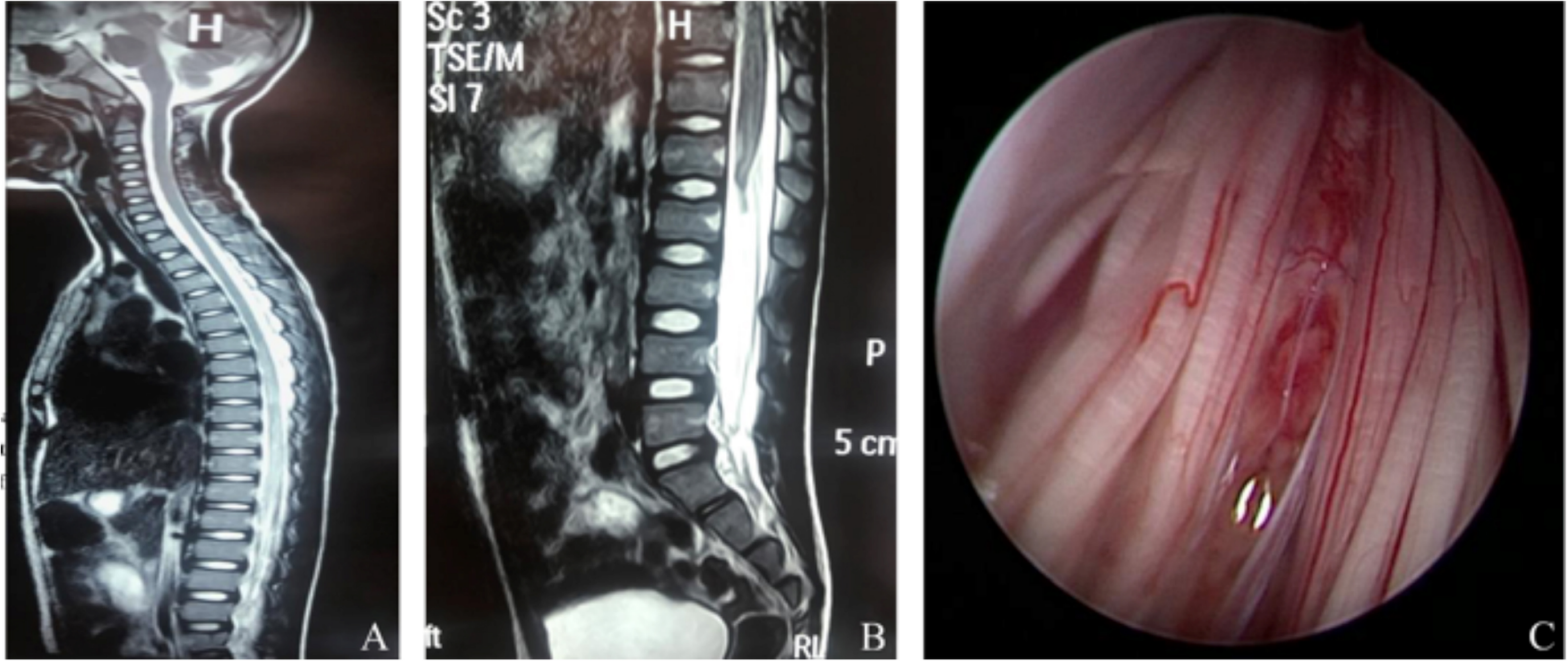
**a** The sagittal MRI images showed longitudinally extended diffused intramedullary patchy T2 hyperintensity at the T12-L1 level. **b** The conus was located at L1 level with mild swelling. **c** Contusion in the conus, terminal filum fibrolipoma and TFT were found in the operation

A healthy 7-year-old boy danced on the bed, making back bend and front bend movements. Suddenly he felt weakness in both of his leg and fell off the bed. He could get up again. About 30 min later, he complained of numbness and weakness in the lower extremities and had incontinence. On admission to the hospital, the patient had paralysis of the lower extremities and dysfunction of two bowel movements. He had no signs or symptoms of tethered cord syndrome prior to the trauma. The patient was placed on a corticosteroid protocol. CT found spina bifida occulta. The sagittal MRI images showed longitudinally extended diffused intramedullary patchy T1 hypointensity and T2 hyperintensity at the T12-L1 level. The conus medullary was located at L1 level with mild swelling. The patient underwent extended surgical exploration and lysis of the filum terminale. Contusion in the conus medullary and 2 mm in diameter TFT with fibrolipoma was found in the operation. The fibrolipoma of the filum terminale was confirmed by histopathology (Fig. 2). Upon discharge, the patient still had paralysis of the lower extremities and incontinence. By the 10-month follow-up, the patient’s left leg muscle strength improved to grade I but still had incontinence and paralysis of his right leg.

### Case three

**Fig. 3:**
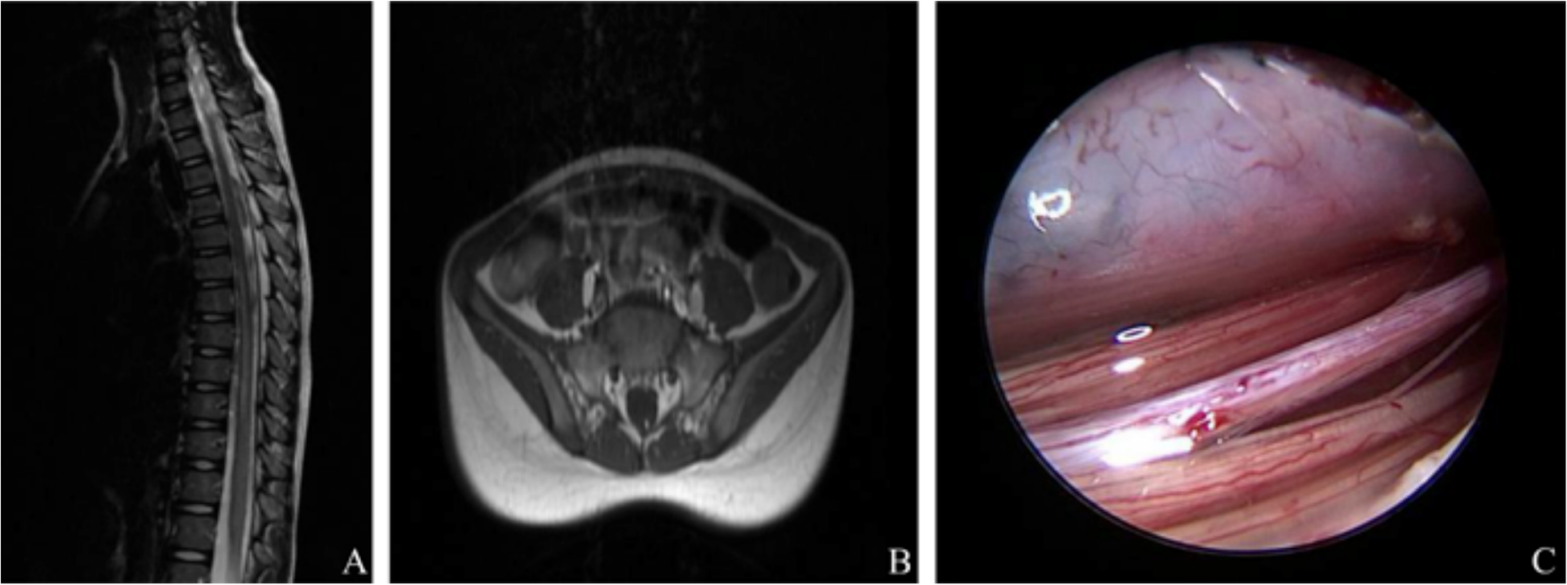
**a** The sagittal MRI images showed longitudinally extended diffused intramedullary patchy T2 hyperintensity at the T6-T10 level. **b** The T1 transverse-sectional MRI of sacral vertebra showed terminal filum fibrolipoma. **c** Terminal filum fibrolipoma and TFT were found in the operation

A healthy 6-year-old girl felt weakness in both legs after performing back bend movement and fell off. She could get up again and had no pain. She had no signs or symptoms of tethered cord syndrome prior to the trauma. About 6 h later, she complained of numbness and weakness in the lower extremities and had incontinence. On admission to the hospital 3 days after injury, the patient had flaccid paraparesis, sensory disturbance below the thoracic dermatome level, and bladder and bowel dysfunction. The sagittal MRI images showed longitudinally extended diffused intramedullary patchy T1 hypointensity and T2 hyperintensity at the T6-T10 level. The conus medullary was located at L1 level. The T1 transverse-sectional MRI of sacral vertebra showed fibrolipoma of the filum terminale. The patient underwent extended surgical lysis of the filum terminale. Swelling in the conus medullary, tortuous dilation of proximal cone vessels, and 4 mm in diameter TFT with fibrolipoma were found in the operation. The fibrolipoma of the filum terminale was confirmed by histopathology (Fig. 3). Upon discharge, the patient still had paralysis of the lower extremities and incontinence. By the 8-month follow-up, the patient still had paralysis of the lower extremities and dysfunction of two bowel movements.

## Discussion and conclusion

SCIWORA is relatively common in children under 8 years old [3]. The severity of SCIWORA was related to the age of onset [4]. Thoracic cord injury accounts for 9.61% of pediatric SCIWORA cases and lumbar cord injury accounts for 1.48% [7]. TCS is a stretch-induced functional disorder of the spinal cor d[8].The filum terminale has clinical significance in its contribution to TCS, a form of occult spinal dysraphis m[5, 9, 10].TFT is not an uncommon condition, but is often overlooked and left untreated due to insufficient diagnostic options [11, 12].

Considering the trauma, it was potentially mild in all patients, but resulted in catastrophic damage of the cord. All three SCIWORA patients presented delayed onset flaccid paraparesis, sensory disturbance below the thoracic dermatome, and bladder and bowel dysfunction after the injury. All of the patients exhibited lower thoracic and lumber spinal injuries and the prognosis was poor. In the first case, TFT with fibrous degeneration, contusion, and laceration in the conus medullary were found in the operation. In the second case, contusion in the conus medullary and TFT with fibrolipoma were found in the operation. In the third case, swelling in the conus medullary, tortuous dilation of proximal cone vessels and 4 mm in diameter TFT with fibrolipoma were found in the operation.

Considering the preexisting spinal or neurological abnormality, this is the first report of SCIWORA with TFT. Preoperative MRI showed fibrolipoma of the filum terminale in the third case. Terminal filum with fibrous degeneration was found in the first case and terminal filum fibrolipoma was found in the second case and the third case. TFT was found in all cases during operation. The basic underlying problem in TFT is abnormal longitudinal stretch on the conus by the filum. In this case a subclinical degree of spinal cord traction is present. The exact pathophysiological mechanisms are not yet completely elucidated. Yamada et al. reported that the longitudinal stretch precipitates metabolic derangements equivalent to ischemic injury [13]. Experimental models studies show that mild to moderate stretch causes transient reductions in metabolism and severe stretch precipitates persistent metabolic derangements that may not recover [14]. The pediatric spine is vulnerable to external forces and allows for significant intersegmental movement [15].. Tani et al. reported that stretch of the terminal filum produces maximum elongation of the lumbo-sacral enlargements in the spinal cor d[16].The mechanism of the trauma could have been related to the flexion–extension movement, which may cause “additional tugging” in congenital tight conus and damage the cord [17–19]. Despite the small number of patients in this report, we inferred that pre-existing TFT might be a predisposing factor for SCIWORA following minor trauma.

In conclusion, we suggest that TFT might be a predisposing factor for SCIWORA and chronic spinal cord traction play an important role in the mechanism of pediatric thoracic and lumber SCIWORA following minor trauma. However, future studies are needed to test its validity. Patients who never undergo treatment for TFT likely have an elevated risk of developing SCIWORA following minor trauma.

## Data Availability

The dataset supporting the conclusions of this article is included within the article.

## Abbreviations

CT: Computed tomography
MRI: Magnetic resonance imaging
SCIWORA: Spinal cord injury without radiographic abnormality
TCS: Tethered cord syndrome
TFT: Tight filum terminale

## References

[1] Pang D, Wilberger JE (1982). Spinal cord injury without radiographic abnormalities in children. J Neurosurg.

[2] Szwedowski D, Walecki J (2014). Spinal cord injury without radiographic abnormality (SCIWORA) - clinical and radiological aspects. Pol J Radiol.

[3] Hendey GW, Wolfson AB, Mower WR, Hoffman JR, National Emergency XRUSG (2002). Spinal cord injury without radiographic abnormality: results of the National Emergency X-radiography utilization study in blunt cervical trauma. J Trauma.

[4] Pang D (2004). Spinal cord injury without radiographic abnormality in children, 2 decades later. Neurosurgery.

[5] Saker E, Henry BM, Tomaszewski KA, Loukas M, Iwanaga J, Oskouian RJ, Tubbs RS (2017). The filum terminale internum and externum: a comprehensive review. J Clin Neurosci.

[6] Bhimani AD, Selner AN, Patel JB, Hobbs JG, Esfahani DR, Behbahani M, Zayyad Z, Nikas D, Mehta AI (2019). Pediatric tethered cord release: an epidemiological and postoperative complication analysis. J Spine Surg.

[7] Carroll T, Smith CD, Liu X, Bonaventura B, Mann N, Liu J, Ebraheim NA (2015). Spinal cord injuries without radiologic abnormality in children: a systematic review. Spinal Cord.

[8] Yamada S, Won DJ (2007). What is the true tethered cord syndrome?. Childs Nerv Syst.

[9] Usami K, Lallemant P, Roujeau T, James S, Beccaria K, Levy R, Di Rocco F, Sainte-Rose C, Zerah M (2016). Spinal lipoma of the filum terminale: review of 174 consecutive patients. Childs Nerv Syst.

[10] Kashlan ON, Wilkinson DA, Morgenstern H, Khalsa SS, Maher CO. Predictors of surgical treatment in children with tethered fibrofatty filum terminale. J Neurosurg Pediatr. 2019:1–8. 10.3171/2019.8.PEDS19292 31675690

[11] O'Neill BR, Gallegos D, Herron A, Palmer C, Stence NV, Hankinson TC, Corbett Wilkinson C, Handler MH (2017). Use of magnetic resonance imaging to detect occult spinal dysraphism in infants. J Neurosurg Pediatr.

[12] Gupta A, Rajshekhar V (2018). Fatty filum terminale (FFT) as a secondary tethering element in children with closed spinal dysraphism. Childs Nerv Syst.

[13] Yamada S, Won DJ, Pezeshkpour G, Yamada BS, Yamada SM, Siddiqi J, Zouros A, Colohan AR (2007). Pathophysiology of tethered cord syndrome and similar complex disorders. Neurosurg Focus.

[14] Tu A, Steinbok P (2013). Occult tethered cord syndrome: a review. Childs Nerv Syst.

[15] Paleologos TS, Fratzoglou MM, Papadopoulos SS, Chatzidakis EE, Gouliamos AD, Kourousis DD (1998). Posttraumatic spinal cord lesions without skeletal or discal and ligamentous abnormalities: the role of MR imaging. J Spinal Disord.

[16] Tani S, Yamada S, Knighton RS (1987). Extensibility of the lumbar and sacral cord. Pathophysiology of the tethered spinal cord in cats. J Neurosurg.

[17] Ren J, Zeng G, Ma YJ, Chen N, Chen Z, Ling F, Zhang HQ (2017). Pediatric thoracic SCIWORA after back bend during dance practice: a retrospective case series and analysis of trauma mechanisms. Childs Nerv Syst.

[18] Wang YJ, Zhou HJ, Wei B, Liu GL, Zheng Y, Zhang Y, Hao CX, Kang HQ, Yuan Y, Lu XL (2016). Clinical characteristics analysis of 120 cases of pediatric spinal cord injury without radiologic abnormality. Zhonghua Yi Xue Za Zhi.

[19] Schneider RC, Cherry G, Pantek H (1954). The syndrome of acute central cervical spinal cord injury; with special reference to the mechanisms involved in hyperextension injuries of cervical spine. J Neurosurg.

